# Effect of training systems on spring frost tolerance of ‘Thompson Seedless’ grapevine

**DOI:** 10.1186/s12870-026-08550-6

**Published:** 2026-04-20

**Authors:** Mohammad Ghasemi, Moazzam Hassanpour Asil, Rouhollah Karimi, Amir Sahraroo

**Affiliations:** 1 Department of Horticultural Science, Faculty of Agriculture, Guilan University, Guilan, Iran; 2 Department of Horticultural Sciences and Landscape Engineering, Faculty of Agriculture, Malayer University, Malayer, Iran

**Keywords:** Antioxidant compounds, Canopy microclimate, Carbohydrate accumulation, Electrolyte leakage, Oxidative stress, Proline content

## Abstract

**Background:**

Grapevine (*Vitis vinifera* L.) is one of the world’s most important fruit crops, but its production is increasingly challenged by climate change and the rising incidence of spring frost, particularly in sensitive cultivars like ‘Thompson Seedless’. The selection of an appropriate trellis training system and the precise timing of management operations, particularly pruning timing, are key strategies for mitigating frost damage. This study aimed to evaluate the interactive effects of year (2023 and 2024 growing seasons), trellis training system (I, T, and Y), and sampling time (March 5, March 25, April 14, and May 5) on the physiological and biochemical indices related to spring frost tolerance in ‘Thompson Seedless’ grapevine.

**Results:**

The study was conducted as a split-plot in time within a randomized complete block design. Analysis of variance revealed that the main effects of year, sampling time, and training system were significant (*p* < 0.01) for all measured indices, including frost tolerance (assessed by electrolyte leakage and budbreak assays), relative water content (RWC), soluble proteins, starch, soluble carbohydrates, proline, malondialdehyde (MDA), hydrogen peroxide (H_2_O_2_), and total phenols. Among the training systems, the Y and T systems demonstrated superior performance in both years. These systems were associated with the highest levels of soluble protein (14.80 and 16.16 mg/g FW, respectively), total phenols (12.25 and 12.07 mg GAE/g FW), and frost tolerance as measured by electrolyte leakage (LT_50_ of Y=-12.68 °C and T=-10.90 °C). Conversely, the I system showed greater sensitivity, marked by increased MDA content (7.34 nmol/g FW) and reduced frost tolerance in the budbreak assay (LT_50_ of -7.23 °C).

**Conclusions:**

The Y and T training systems enhance spring frost tolerance in ‘Thompson Seedless’ grapevine by improving the antioxidant defense system and promoting the accumulation of osmolytes. Our findings suggest that the Y and T systems, respectively, are the most effective treatments for improving spring frost tolerance by enhancing the physiological status and reducing oxidative stress in grapevine buds. These systems are recommended as a sustainable management strategy in vineyards prone to spring frost.

## Introduction

Grapevine (*Vitis vinifera* L.) is one of the world’s most economically and nutritionally significant fruit crops, consumed both fresh and as processed products [1]. According to the International Organisation of Vine and Wine (OIV), the global vineyard area was approximately 7.1 million hectares in 2024, with an annual fresh grapeproduction of around 77.7 million tons, ranking among the highest for fruit crops [2]. Europe, Asia and Americas are the primary grape-growing regions, collectively accounting for over 95% of the global vineyard area Spain (13%), France (11%), China (11%), Italy (10%), and Turkey (6%) are the leading countries by cultivation area, collectively accounting for about 51% of the world’s vineyards [2]. Iran with an annual production of approximately 1.5 million tons in 2023, ranks around twelfth to fourteenth globally in production and is a major exporter of raisins [3, 4]. The cultivar ‘Thompson Seedless’ is widely cultivated in semi-arid regions such as Iran, California, and the Mediterranean due to its desirable traits, including being seedless, mid-season ripening, high and stable yield, excellent drying quality, high marketability, and adaptability to various climatic conditions [5]. However, in the face of accelerating climate change, the vulnerability of sensitive cultivars like ‘Thompson Seedless’ to spring frost events has emerged as a major constraint on global viticulture productivity, potentially leading to yield losses of up to 50–90% in affected regions [6–8]. This study addresses this gap by evaluating training systems as a low-cost, sustainable adaptation strategy, which is crucial for maintaining economic viability in frost-prone areas and ensuring food security amid shifting climate patterns. However, global warming and climate change have increased the risk of spring frost, which highlights the critical role of grapevine training systems as a key vineyard management practice for climate change adaptation [6, 7, 9]. Climate change, particularly the increased frequency and intensity of late spring frosts, poses a serious threat to viticulture by causing severe damage to young shoots, buds, and inflorescences, thereby substantially reducing yield in sensitive cultivars such as ‘Thompson Seedless’ [10]. In temperate regions such as parts of Iran, the Mediterranean, and California, late spring frosts have become more unpredictable, impacting grapevine performance [11, 12]. Grapevine yield is highly sensitive to environmental factors, especially temperature fluctuations during critical phenological stages like budbreak and flowering [9]. Recent studies have shown that climate change has caused an overlap between sensitive growth stages and high-risk periods for late frosts, increasing the need to reconsider the choice of training systems [13]. In this context, training systems must not only be adapted to the regional climate but also be capable of modifying the microclimate and delaying budbreak to prevent spring frost damage. Particularly in semi-arid regions, the choice of an appropriate training system can make a significant difference in bud survival and yield preservation [14]. Furthermore, research indicates that training systems that allow for the adjustment of canopy structure and the height of the fruiting zone play a crucial role in mitigating risks associated with temperature inversions [15]. Spring frost damage in grapevines induces profound physiological disruptions, primarily through extracellular ice formation that causes cellular dehydration, membrane rupture, and loss of tissue viability [8, 10, 16]. This leads to increased electrolyte leakage as a marker of membrane integrity, often assessed via LT50 values, which directly correlates with reduced bud survival and shoot development [16, 17]. Additionally, frost impairs hydraulic conductivity by inducing xylem embolism, where air bubbles block water transport vessels, exacerbating post-frost wilting and limiting nutrient uptake [12, 18, 19]. Carbon reserves, such as starch and soluble carbohydrates stored in buds and canes, are critically depleted during frost recovery, as vines mobilize these osmolytes for cryoprotection and repair, potentially compromising subsequent growth and yield if reserves are insufficient [8, 20, 21]. Oxidative stress from reactive oxygen species (ROS) accumulation further aggravates damage, elevating malondialdehyde (MDA) and hydrogen peroxide (H_2_O_2_) levels, while antioxidants like phenols and osmolytes such as proline mitigate these effects [16, 18]. These well-documented consequences justify our selection of parameters: electrolyte leakage and budbreak assays for tissue viability; relative water content (RWC), MDA, and H_2_O_2_ for membrane and oxidative stress; starch, soluble carbohydrates, and proline for carbon reserves and osmoprotection; and total phenols for antioxidant defense. By linking training systems to these indices, this study provides mechanistic insights into frost tolerance enhancement. Various methods exist to mitigate the effects of cold and enhance frost tolerance. Cold hardiness varies depending on the cultivar, species, rootstock, genetics, and phenological stage, and is also influenced by climatic conditions and viticultural practices such as training systems, delayed pruning, irrigation, nutrition, and crop load [18].Training systems, by balancing the light received by the canopy, have a significant effect on carbohydrate storage in canes and buds and on tissue maturation. Adequate light during the growing season increases frost tolerance, as high light levels within the vine canopy are associated with greater storage of soluble carbohydrates [12, 20, 21]. These systems influence shoot architecture, affecting light interception, air circulation, and overall plant physiology, and can also regulate the timing of phenological events like budbreak and flowering [22–26]. For instance, systems with a higher trunk height (such as high cordon or pergola) can reduce spring frost damage by moving the fruiting zone away from the cold air layers near the ground and creating a narrower daily temperature range. In this framework, recent studies have increasingly emphasized that grapevine training systems influence spring frost tolerance not only through phenological effects such as delayed budbreak, but also by modifying canopy microclimate, light interception efficiency, and carbon assimilation [14, 22]. Improved light distribution within the canopy enhances carbohydrate reserve accumulation and antioxidant capacity, which together stabilize cellular membranes and reduce oxidative damage during freeze–thaw stress [18, 27, 28]. Consequently, training systems indirectly regulate cold-stress response pathways by linking canopy architecture with physiological mechanisms of frost tolerance [14, 29].This approach raises minimum temperatures and lowers maximum temperatures without significantly changing the mean seasonal temperature, which is beneficial for protecting buds during temperature inversions [15, 30]. Common training systems like Vertical Shoot Positioning (VSP), Geneva Double Curtain (GDC), and the Lyre system have different effects on the plant microclimate and phenology. The VSP system creates a compact canopy and increases light penetration but may exacerbate frost risk at lower heights, whereas sprawling systems allow for free growth but can reduce light penetration [22]. Given the high plasticity of grapevines, a variety of training and guidance methods exist, with the choice depending on the cultivar, end-use, climatic conditions, potential for mechanization, and economic factors. The vine training system alters cold hardiness by influencing cane vigor and the specific microclimate of each vine; proper canopy management can enhance this tolerance. Additionally, trellis systems that direct canes horizontally or downwards help prevent the return of elaborated sap to the roots and increase the sugar content of the fruit, which also benefits the following year’s yield [31]. Field experiments have demonstrated that trellis training systems optimize the leaf area to fruit weight ratio, enhancing source-sink balance and promoting the allocation of carbohydrates from vegetative to reproductive organs [5, 22, 32]. Additionally, canopy management can indirectly influence nitrogen status and carbon-to-nitrogen (C/N) ratios [19]. Despite the widespread use of these systems, their comparative effects on the timing of budbreak and spring frost tolerance in ‘Thompson Seedless’ grapevine, especially in semi-arid regions prone to late spring frosts like Iran, have been less studied. Therefore, this study aimed to evaluate and compare the effects of I, T, and Y bilateral cordon training systems on physiological and biochemical traits associated with spring frost tolerance in ‘Thompson Seedless’ grapevine, with the goal of identifying the most resilient training configuration for frost-prone, semi-arid regions thereby advancing adaptive viticultural practices under climate change.

## Materials and methods

### Plant materials and experimental site

This research was conducted as a split-plot in time within a randomized complete block design over two years (2023–2024) at the research vineyard of the Grape and Raisin Research Institute of Malayer University, located in Hamadan province, Iran. The geographical coordinates of the vineyard center were 301,661 E and 3,793,596 N (UTM Zone 39 S). Only commercial and widely used grape varieties are planted in this vineyard and there are no wild grape varieties or genotypes, no permits were required. The study aimed to evaluate the effect of Three bilateral cordon-trained trellis systems were evaluated: I system (vertical cordon with shoots trained upward), T system (horizontal cordon with shoots trained vertically), and Y system (bilateral cordon arms angled upward forming a Y-shape with shoots trained vertically) on the spring frost tolerance of 8-year-old own-rooted ‘Thompson Seedless’ (*Vitis vinifera* L.) grapevines. All cuttings were collected from these own-rooted vines, ensuring uniformity in plant material origin and eliminating potential confounding effects from rootstock. The main plot factor was the three trellis systems (I, T, and Y), and the sub-plot factor was four sampling times (March 5, March 25, April 14, and May 5, 2023). The experiment was conducted with three blocks (replications). Each block contained all three training system treatments as main plots, and vines within each training system were sampled at four different time points as sub-plots. Vines were spaced 2 m apart within rows and 3 m between rows. Irrigation was applied via a drip system with a 10-day cycle. Standard viticultural practices for the region, including nutrition, pruning, and pest and disease management, were performed as needed. All measurements of the studied traits were carried out in the specialized laboratories of the Faculty of Agriculture at the University of Guilan and Malayer University.

### Post-freezing budbreak assay (PFBA) and Electrolyte Leakage (EL) Assay

Spring frost tolerance was evaluated using the PFBA. Briefly, five uniform cuttings (15–20 cm in length and 8–12 mm in diameter, each containing at least three healthy buds from nodes 4–8 of one-year-old canes) were collected from around each vine at each sampling stage and transferred to the laboratory. A specific number of cuttings were assigned to each freezing treatment (-4, -6, -10, -14, and − 18 °C) in a programmable freezer (Rad Electronic, Tehran, Iran). After freezing, the cuttings were placed in moist sand at 6 °C and 90% relative humidity for one week to complete their chilling requirement. Subsequently, the cuttings were planted in plastic pots containing a perlite: peat moss mixture (3:1 v/v) and maintained in a research greenhouse at 20/15°C (day/night). The evaluation criterion was the emergence of the terminal bud after 30 days. The percentage of budbreak was calculated by dividing the number of cuttings with green buds by the total number of cuttings [16].

For El measurement, after the freezing treatments, canes were removed from the freezer and allowed to thaw gradually, first for 2 h at 4 °C and then for 3 h at room temperature (25 °C). From each temperature treatment, five buds were sharply excised and individually immersed in 70 mL vials containing 40 mL of distilled water. The vials were placed on a shaker at 120 rpm for 20 h at room temperature. The initial electrical conductivity (EC1) was measured using a conductivity meter (Atago, Japan). The tubes were then autoclaved for 20 min at 121 °C. After cooling, the final electrical conductivity (EC2) was measured [33]. The relative electrolyte leakage (REL) was calculated using the formula:

REL (%) = (EC1 / EC2) × 100.

### Relative water content (RWC)

The RWC of buds was measured using the method described by Smart & Bingham (1974). At each sampling stage, one-year-old canes were collected, and buds from the middle nodes were detached. Fresh weight (FW) was recorded immediately. Turgid weight (TW) was measured after 24 h of immersion in distilled water at room temperature (25 °C) in the dark. Dry weight (DW) was determined after drying in an oven at 70 °C for 72 h [34]. RWC was calculated using the formula:

RWC (%) = [(FW - DW) / (TW - DW)] × 100.

### Soluble proteins and proline

Soluble proteins were measured according to the Bradford (1976) method. A 0.5 g sample of fresh bud tissue was homogenized in 5 mL of extraction buffer (50 mM Tris-HCl, pH 7.8, containing 1 mM EDTA and 1% PVP) and centrifuged at 12,000 × g for 20 min at 4 °C. A 50 µL aliquot of the supernatant was mixed with 1 mL of Coomassie Brilliant Blue G-250 reagent. After 5 min of incubation at room temperature, the absorbance was read at 595 nm. Protein concentration was determined using a standard curve of bovine serum albumin (BSA) and expressed as mg/g fresh weight (FW) [35].

Proline concentration was determined according to the method of Bates et al. (1973). A 0.5 g sample of bud tissue was homogenized in 10 mL of 3% (w/v) sulfosalicylic acid. Two mL of the extract was mixed with 2 mL of glacial acetic acid and 2 mL of ninhydrin reagent. The mixture was heated for 1 h at 100 °C. After cooling, 4 mL of toluene was added and vortexed. The absorbance of the toluene phase was read at 518 nm. Proline concentration was calculated from a standard curve and expressed as µmol/g FW [36].

### Starch and soluble carbohydrates

Starch was extracted and measured based on the method of Hedge and Hofreiter (1962) with minor modifications. A 0.5 g sample of bud tissue was homogenized with 5 mL of 80% (v/v) ethanol and centrifuged. The pellet was washed three times with 80% ethanol. The residue was mixed with 5 mL of distilled water and 6.5 mL of 52% (w/w) perchloric acid. After 15 min, the mixture was centrifuged, and the supernatant was collected. The extraction was repeated. The final volume was brought to 100 mL with distilled water [37]. A 0.2 mL aliquot was mixed with 3 mL of fresh anthrone reagent. The tubes were heated in a boiling water bath for 7.5 min and then cooled in ice. Absorbance was read at 630 nm. Starch concentration was calculated from a glucose standard curve and expressed as mg/g FW.

Soluble carbohydrates were measured by the method of Yemm and Willis (1954) with minor modifications. Dried bud samples (0.5 g) were homogenized in 80% (v/v) ethanol and centrifuged. The extraction was repeated twice with 70% ethanol. A 0.1 mL aliquot of the alcoholic extract was mixed with 3 mL of fresh anthrone reagent, heated in a boiling water bath for 15 min, and cooled in ice. Absorbance was read at 630 nm. Carbohydrate concentration was calculated from a glucose standard curve and expressed as mg/g dry weight (DW) [38].

### MDA and H_2_O_2_

Lipid peroxidation was estimated by measuring the concentration of MDA according to the method of Heath and Packer (1968) with minor modifications. A 0.5 g sample of fresh bud tissue was homogenized in 5 mL of 0.1% (w/v) trichloroacetic acid (TCA) and centrifuged. One mL of the supernatant was mixed with 1 mL of 0.5% thiobarbituric acid (TBA) in 20% TCA. The mixture was heated at 95 °C for 30 min and then cooled. After centrifugation, the absorbance of the supernatant was read at 532 nm and corrected for non-specific absorbance at 600 nm. MDA concentration was calculated using an extinction coefficient of 155 mM⁻¹ cm⁻¹ and expressed as nmol/g FW [39].

H_2_O_2_ content was measured according to the method of Alexieva et al. (2001) with minor modifications. A 0.5 g sample of fresh bud tissue was homogenized in 5 mL of 0.1% (w/v) TCA and centrifuged. A 0.5 mL aliquot of the supernatant was mixed with 0.5 mL of 10 mM potassium phosphate buffer (pH 7.0) and 1 mL of 1 M potassium iodide (KI). After 10 min of incubation in the dark, the absorbance was read at 390 nm. H_2_O_2_ concentration was calculated from a standard curve and expressed as µmol/g FW [40].

### Total phenols

Total phenols were extracted and measured based on the method of Velioglu et al. (1998) with slight modifications. A 0.5 g sample of frozen bud tissue was homogenized in 5 mL of 80% (v/v) methanol containing 1% HCl. The mixture was shaken for 2 h in the dark at 4 °C and centrifuged. A 100 µL aliquot of the supernatant was mixed with 500 µL of diluted Folin-Ciocalteu reagent (1:10) and 400 µL of 7.5% (w/v) sodium carbonate. The final volume was brought to 2 mL with distilled water. After 90 min of incubation in the dark, absorbance was read at 765 nm. Total phenol content was calculated from a gallic acid standard curve and expressed as mg gallic acid equivalents (GAE)/g FW [41].

### Statistical analysis

Data analysis was performed using SAS software version 9.4 (M8). Mean comparisons were conducted using Tukey’s Honestly Significant Difference (HSD) test at the 1% probability level, and graphs were generated using Microsoft Excel. Cold hardiness was evaluated using the LT_50_ index, defined as the temperature causing 50% electrolyte leakage. LT_50_ values were estimated by fitting a logistic regression model to the electrolyte leakage data across freezing temperatures, and calculations were performed using Microsoft Excel.

## Results

### Frost tolerance as estimated by PFBA and EL method

Frost tolerance assessed by the PFBA method was significantly influenced by year, sampling date, and training system (Table 1). Overall, frost tolerance was higher in the second year of the study compared with the first year. Among training systems, the Y system consistently exhibited the highest frost tolerance, followed by the T system, whereas I system showed the lowest tolerance (Table 2). In all training systems, frost tolerance progressively declined from early March to May.

**Table 1:** Analysis of variance for the effects of year, training system, sampling date, and their interactions on physiological and biochemical indices in buds of ‘Thompson Seedless’ grapevine

Source of variance	DF	Mean square				
		PFBA LT_50_	EL LT_50_	RWC	Soluble protein content	Starch content
Y (Year)	1	16.75^**^	48.65^**^	3.85^ns^	116.17^**^	213.38^ns^
B (Y) (block (Year))	4	0.58^ns^	0.20^ns^	40.37^**^	1.18^**^	20.65^ns^
S (Training system)	2	60.46^**^	55.80^**^	48.49^**^	87.22^**^	1462.28^**^
Y*S	2	0.02^ns^	0.012^ns^	2.38^ns^	0.004^ns^	2.13 ^ns^
S*B (Y)^†^	8	0.09	0.09	3.04	0.01	49.61
T (Time)	3	681.62^**^	682.76^**^	1732.9^**^	42.04^**^	1833.26^**^
Y*T	3	0.12^ns^	0.18^ns^	6.96^ns^	0.003^ns^	2.41^ns^
T*S	6	0.01 ^ns^	0.14 ^ns^	2.58^ns^	0.005^ns^	313.81^**^
Y*T*S	6	0.09^ns^	0.10^ns^	3.91^ns^	0.005^ns^	12.18^ns^
Error	36	0.26	0.29	4.22	0.15	58.69
CV	-	-5.90	-4.86	2.90	2.75	13.58

** *p* < 0.01; *ns* not significant, *DF* degrees of freedom, *EL LT*₅₀ lethal temperature causing 50% mortality or injury assessed by electrolyte leakage (EL), *PFBA LT*₅₀, lethal temperature for 50% mortality assessed by post-freezing budbreak assay, *RWC* relative water content, *FW* fresh weight.

Negative sign for CV in LT₅₀ columns is due to negative mean values.

^†^ The S × Block (Year) term was used as the error term for testing training system effects

**Table 2:** Main effects of year, training system, and sampling date on physiological and biochemical indices in grapevine

Treatment levels	PFBA LT_50_ (°C)	EL LT_50_ (°C)	RWC (%)	Soluble protein content (mg/g FW)	Starch content (mg/g FW)
Year					
1 (2023)	-8.20 ^a^	-10.25 ^a^	not shown due to ns effect	13.18 ^b^	` not shown due to ns effect
2 (2024)	-9.16 ^b^	-11.90 ^b^		15.72 ^a^	
Training system (S)					
I	-7.23 ^a^	-9.64 ^a^	69.20 ^c^	12.40 ^c^	48.72 ^a^
T	-8.43 ^b^	-10.90 ^b^	72.04 ^a^	14.80 ^b^	58.18 ^b^
Y	-10.37 ^c^	-12.68 ^c^	70.71 ^ab^	16.16 ^a^	64.33 ^b^
Sampling date (T)					
March 5	-15.58 ^d^	-17.92 ^d^	62.09 ^c^	15.12 ^b^	67.12 ^a^
March 25	-11.94 ^c^	-14.39 ^c^	63.56 ^c^	16.04 ^a^	62.21 ^a^
April 14	-4.98 ^b^	-7.43 ^b^	73.87 ^b^	14.20 ^c^	51.38 ^b^
May 5	-2.22 ^a^	-4.55 ^a^	83.09 ^a^	12.46 ^d^	44.93 ^b^

Means within each main effect followed by different letters are significantly different according to Tukey’s HSD test (*p* < 0.01).

*EL LT*_50_ lethal temperature causing 50% mortality or injury assessed by electrolyte leakage (EL), *PFBA LT*₅₀ lethal temperature for 50% bud mortality assessed by post-freezing budbreak, *RWC* relative water content, *FW* fresh weight

EL based frost tolerance showed significant effects of year, sampling date, and training system (Table 1). Similar to PFBA results, the Y training system exhibited the greatest membrane stability (lowest LT_50_ values), followed by the T system, while I system was the most sensitive (Table 2). Higher frost tolerance was observed in the second year of the experiment.

### RWC

Bud relative water content (RWC) was significantly affected by sampling date and training system (Table 1). The T and Y training systems generally maintained higher RWC values compared with I system (Table 2). Across sampling dates, RWC showed an increasing trend toward early May.

### Soluble proteins and proline

Soluble protein content in buds was significantly influenced by year, sampling date, and training system (Table 1). Higher protein accumulation was observed in the second year of the study. Among training systems, the Y system showed the highest soluble protein content, particularly during early spring sampling dates (Table 2).

Proline content was significantly affected by sampling date, training system, and their interaction (Table 3). Proline accumulation peaked during early spring, particularly at the March 5 sampling date. Among training systems, I system exhibited the highest proline content, especially at early sampling stages (Table 4).

**Table 3:** Interaction effects of sampling date × training system on physiological and biochemical indices in grapevine

Sampling date	Training system	Proline (µmol/g FW)	Starch content (mg/g FW)
March 5	I	8.09^a^	58.75^bcde^
	T	7.14^b^	67.55^abc^
	Y	6.27^c^	75.05^a^
March 25	I	4.29^d^	53.39^cdef^
	T	4.29^d^	62.15^abcd^
	Y	3.56^e^	71.09^ab^
April 14	I	3.3^ef^	34.62^g^
	T	2.87^fg^	54.15^cdef^
	Y	2.59^g^	65.35^abc^
May 5	I	2.37^g^	48.12^defg^
	T	1.61^h^	40.85^fg^
	Y	1.31^h^	45.81^efg^

Means within columns followed by different letters are significantly different according to Tukey’s honestly significant difference (HSD) test (*p* < 0.01).

*FW* fresh weight

**Table 4:** Analysis of variance for the effects of year, training system, sampling date, and their interactions on antioxidant defense and osmolyte indices in buds of ‘Thompson Seedless’ grapevine

Source of Variance	DF	Mean square				
		Soluble carbohydrates	Proline	Malondialdehyde	Hydrogen peroxide	Total phenolic
Y(year)	1	68.81 ^ns^	0.66^ns^	2.43^**^	4.25^**^	241.96^**^
B(Y) (block(year))	4	284.67^**^	0.37^**^	1.43^**^	0.32^*^	2.37^**^
S*B(Y)^†^	8	4.68	0.036	0.15	0.127	0.002
S(Training system)	2	279.38^**^	13.58^**^	5.78^**^	3.78^**^	27.91^**^
Y*S	2	4.20^ns^	0.01^ns^	0.005^ns^	0.05^ns^	0.001^ns^
T(Time)	3	16091.4^**^	93.58^**^	29.24^**^	8.81^**^	122.11^**^
Y*T	3	25.36^ns^	0.11^ns^	0.10^**^	0.19^ns^	0.004^ns^
T*S	6	17.36^ns^	0.18^*^	0.04^ns^	0.11^ns^	0.006 ^ns^
Y*T*S	6	1.35^ns^	0.11^ns^	0.01^ns^	0.05^ns^	0.006^ns^
Error	36	16.63	0.06	0.022	0.08	0.043
CV	-	6.31	6.65	2.16	5.66	1.81

** *p* < 0.01; * *p* < 0.05; ns, not significant. DF, degrees of freedom.

^†^ The S × Block (Year) term was used as the error term for testing training system effects

### Starch and soluble carbohydrates

Starch content was significantly affected by sampling date and its interaction with training system (Table 1). Higher starch accumulation occurred during early sampling dates, particularly in March. The Y training system exhibited the greatest starch reserves at early spring, especially at the March 5 sampling date (Table 4).

Soluble carbohydrate content was significantly influenced by sampling date and training system, with no significant interaction between these factors (Table 3). The Y and T training systems consistently showed higher carbohydrate accumulation compared with the I system. Maximum soluble carbohydrate levels were recorded in late March (Table 5).

**Table 5:** Main effects of year, training system, and sampling date on antioxidant defense and osmolyte indices in grapevine

Treatment levels	Soluble carbohydrates (mg/g DW)	Proline (µmol/g FW)	Malondialdehyde (nmol/g FW)	Hydrogen peroxide (µmol/g FW)	Total phenolic (mg/g FW)
Year					
1 (2023)	not shown due to ns effect		6.78 ^a^	4.94 ^a^	9.71 ^b^
2 (2024)			7.14 ^a^	5.43 ^a^	13.37 ^a^
Training system (S)					
I	60.71 ^b^	4.75 ^a^	7.34 ^a^	5.57 ^a^	10.30 ^c^
T	66.07 ^a^	3.90 ^b^	7.13 ^a^	5.21 ^a^	12.25 ^a^
Y	67.04 ^a^	3.26 ^c^	6.41 ^b^	4.78 ^b^	12.07 ^b^
Sampling date (T)					
March 5	31.94 ^c^	7.16 ^a^	5.48 ^d^	4.51 ^d^	13.45 ^a^
March 25	63.09 ^b^	3.57 ^b^	6.56 ^c^	4.86 ^c^	13.01 ^b^
April 14	104.38 ^a^	3.39 ^b^	8.51 ^a^	6.14 ^a^	11.95 ^c^
May 5	59.03 ^b^	1.76 ^c^	7.30 ^b^	5.24 ^b^	7.75 ^d^

Means within each main effect followed by different letters are significantly different according to Tukey’s honestly significant difference (HSD) test (*p* < 0.01).

*DW* dry weight, *FW* fresh weight

### Total phenols

Total phenolic content was significantly influenced by year, sampling date, and training system (Table 3). Higher phenolic accumulation was observed in the second year of the study. Among training systems, the T system exhibited the greatest total phenolic content, while peak values were generally observed during early spring sampling dates (Table 5).

### H_2_O_2_ and MDA content

H₂O₂ content was significantly affected by year, sampling date, and training system, with no significant interaction effects (Table 3). Higher H₂O₂ accumulation was observed in I system, while the T and Y systems maintained lower levels across sampling dates (Table 5).

MDA content was significantly influenced by year, sampling date, and training system (Table 3). Higher MDA levels were observed in the second year and at mid-spring sampling dates, indicating increased lipid peroxidation. Among training systems, I system exhibited higher MDA accumulation, whereas the T and Y systems showed lower levels (Tables 5 and 6).

**Table 6:** Interaction effects of year × sampling date on malondialdehyde content in buds of ‘Thompson Seedless’ grapevine

Year	Sampling date	Malondialdehyde (nmol/g FW)
1 (2023)	March 5	5.36 ^h^
	March 25	6.35 ^f^
	April 14	8.23 ^b^
	May 5	7.18 ^d^
2 (2024)	March 5	5.60 ^g^
	March 25	6.77 ^e^
	April 14	8.79 ^a^
	May 5	7.43 ^c^

Means followed by different letters within the column are significantly different according to Tukey’s honestly significant difference (HSD) test (*p* < 0.01).

*FW* fresh weight, *MDA* malondialdehyde

## Discussion

Our results show that bilateral cordon Y and horizontal T training systems significantly enhance spring frost tolerance of ‘Thompson Seedless’ compared with the vertical I system. This conclusion is supported by both post-freezing budbreak assay (PFBA) and electrolyte-leakage based LT₅₀ estimates (Y: PFBA/EL LT₅₀ = −10.37/−12.68°C; T: −8.43/−10.90°C; I: −7.23/−9.64°C; Tables 2 and 5). The advantage of Y and T was consistent across years and sampling dates, although absolute tolerance declined from early March toward May in all systems.

The superior frost tolerance of Y and T systems co-occurred with multiple physiological changes known to mitigate freezing damage. Y and T buds had higher soluble protein (Y: 16.16 mg g⁻¹ FW; T: 14.80 mg g⁻¹ FW vs. I: 12.40 mg g⁻¹ FW) and total phenolic contents (T: 12.25 mg GAE g⁻¹ FW; Y: 12.07 mg GAE g⁻¹ FW vs. I: 10.30 mg GAE g⁻¹ FW) and greater carbohydrate reserves (starch and soluble sugars) compared with I (Tables 2, 3, 4 and 5). Concurrently, Y and T buds showed lower oxidative damage indicators (MDA: 6.41–7.13 nmol g⁻¹ FW; H₂O₂: 4.78–5.21 µmol g⁻¹ FW vs. I: 7.34 nmol g⁻¹ FW and 5.57 µmol g⁻¹ FW). These coordinated changes directly link the measured biochemical parameters to frost tolerance, as higher carbohydrate availability enhances osmotic adjustment and cryoprotection, while increased protein and phenolic pools stabilize membranes and strengthen antioxidant defenses during freeze–thaw stress Together, these changes explain the lower membrane leakage and improved bud survival observed in Y and T systems [28, 42].

Mechanistically, the Y and T trellis architectures modify canopy geometry and microclimate to favor improved maturation and reserve accumulation. By improving light distribution and reducing shading within the canopy, these systems increase carbon assimilation and promote thicker canes and better periderm development that favor carbohydrate storage [14, 29]. Moreover, reduced apical dominance and altered source–sink relations in Y and T systems promote greater allocation of assimilates toward perennial storage tissues, a mechanism widely associated with enhanced cold hardiness in grapevine and other woody perennials [14, 29].While this study utilized own-rooted ‘Thompson Seedless’ vines, it is worth noting that rootstocks can significantly influence scion frost tolerance by altering hydraulic conductivity [43, 44], carbohydrate partitioning [45, 46], and stress signaling (e.g., ABA-mediated pathways) [47, 48]; Thus, although rootstock effects were intentionally excluded here to isolate training-system responses, future studies should explicitly evaluate rootstock × training system interactions to extend these findings to grafted commercial vineyards [43, 49].

Moreover, altered canopy ventilation and reduced cold-air pooling near the fruiting zone raise minimum temperatures during inversion events, reducing bud exposure to the coldest micro-layer near the soil surface. This microclimatic buffering provides a physical complement to the biochemical advantages observed in Y and T systems, jointly explaining their superior LT₅₀ values.

Sampling-date effects indicate a clear de-acclimation trend: buds sampled in early March were most tolerant (lowest LT₅₀) with peak starch and soluble carbohydrate stocks, whereas tolerance fell markedly by mid-April and May. This decline coincides with bud metabolic reactivation, cambial activity and cell expansion that increase free-water fraction and vulnerability to ice formation. The simultaneous depletion of carbohydrate reserves and rise in oxidative stress markers during this period highlights the central role of carbon status in maintaining antioxidant capacity during de-acclimation. The peak in oxidative stress markers (MDA, H₂O₂) at mid-spring reflects increased metabolic rates and ROS production during de-acclimation/active growth, which can overwhelm antioxidant defenses if carbohydrate and phenolic pools are insufficient. Thus, the temporal pattern supports the mechanistic link between reserve status, oxidative balance, and cold tolerance.

Inter-annual differences were significant: the second year showed overall higher frost tolerance and larger protein/phenolic pools (Tables 2 and 5). This likely reflects climatic differences between seasons (e.g., cumulative radiation, warm/cold spells, and timing of phenology) and their effect on carbon gain and cane maturation in the preceding months. Such inter-annual variability underscores that training-system benefits are modulated by seasonal climatic context rather than acting in isolation.

Our findings align with reports that improved light penetration and canopy architecture increase carbohydrate reserves and antioxidant capacity, thereby enhancing cold tolerance [28, 29]. However, a key discrepancy requires explicit treatment: in our data, I system had the highest proline concentrations (Table 4) yet was the most frost-sensitive. This apparent contradiction highlights the context-dependent role of proline in stress physiology. Two non-exclusive interpretations reconcile this: (1) stress-indication hypothesis - proline accumulation here may largely be a symptom of severe stress (i.e., tissues under greater damage and osmotic imbalance synthesize more proline), not an effective protective adjustment; (2) threshold/compartmentation hypothesis — proline can be protective only when coordinated with sufficient carbohydrate reserves and antioxidant capacity; without those co-factors, elevated proline alone cannot prevent membrane peroxidation. Similar context-dependent roles for proline have been reported in grapevine literature [18, 42]. We therefore interpret the high proline in I as an indicator of heightened stress response rather than proof of superior acclimation in that system.

Practically, our results recommend the adoption of Y or T bilateral cordon systems in ‘Thompson Seedless’ vineyards at risk of late spring frosts in semi-arid regions. These systems provide a low-cost, management-oriented approach to increase bud frost survival via improved microclimate, carbohydrate storage and antioxidant capacity. For growers, integrating Y/T training with complementary measures (careful pruning timing, canopy management, irrigation and nutrition aimed at maximizing pre-winter carbohydrate accumulation) will likely yield the best outcome. From a research perspective, future work should prioritize (i) multi-season and multi-site validation, (ii) integration of hormonal (ABA, auxin, cytokinin) and C/N signaling analyses, and (iii) systematic evaluation of rootstock × training system interactions and long-term impacts on vine vigor and yield stability. In conclusion, Y and T training systems confer reproducible physiological advantages that translate into improved spring frost tolerance in ‘Thompson Seedless’, but their efficacy remains influenced by seasonal climate and management context.

## Conclusions

This study comprehensively demonstrated that the three-way interaction of year, training system, and sampling time plays a pivotal role in enhancing spring frost tolerance and regulating budburst timing in ‘Thompson Seedless’ grapevines. Based on two-year data, the Y and T bilateral cordon training systems emerged as superior, significantly improving plant resistance through enhanced membrane stability (reduced ion leakage), increased accumulation of protective compounds (soluble proteins, total phenols, and soluble carbohydrates), and an improved LT₅₀ index (Y: PFBA/EL LT₅₀ = −10.37/−12.68°C; T: −8.43/−10.90°C). These physiological advantages translated into consistently greater post-freezing bud survival across sampling dates. From a physiological perspective, buds achieved maximum potential tolerance in early stages (March 5) with peak accumulation of starch, soluble carbohydrates, and proline, whereas mid-stages (April 14) exhibited increased oxidative stress, elevating MDA and H_2_O_2_ levels. The concurrent decline in LT₅₀ values during later sampling dates reflects the progression of de-acclimation and increasing metabolic activity in developing buds. Ultimately, we recommend implementing Y and T systems as integrated management strategies in vineyards prone to spring frost, such as in Iran, coupled with irrigation and nutrition management based on terrain slope to maximize productivity and yield. Adoption of such structurally adaptive training systems offers a cost-effective, long-term approach to mitigating climate-related frost risks. This approach ensures sustainable production and contributes to global climate change adaptation goals. Future research could focus on genetic modeling, integration with resistant rootstocks, and multi-season studies across climatic gradients to evaluate long-term effects.

## Data Availability

All data generated or analyzed during this study are included in this published article.

## Abbreviations

BSA: Bovine Serum Albumin
DW: Dry Weight
EC: Electrical Conductivity
EL: Electrolyte Leakage
FAO: Food and Agriculture Organization
FW: Fresh Weight
GAE: Gallic Acid Equivalents
H_2_O_2_: Hydrogen Peroxide
LT_50_: Lethal Temperature for 50% of the population
MDA: Malondialdehyde
PFBA: Post-Freezing Budbreak Assay
REL: Relative Electrolyte Leakage
ROS: Reactive Oxygen Species
RWC: Relative Water Content
TBA: Thiobarbituric Acid
TCA: Trichloroacetic Acid
VSP: Vertical Shoot Positioning
